# Heterodimerization of apelin receptor and neurotensin receptor 1 induces phosphorylation of ERK_1/2_ and cell proliferation *via* Gαq-mediated mechanism

**DOI:** 10.1111/jcmm.12404

**Published:** 2014-08-28

**Authors:** Bo Bai, Xin Cai, Yunlu Jiang, Emmanouil Karteris, Jing Chen

**Affiliations:** a Neurobiology Institute, Jining Medical UniversityJining, Shandong, China; b Biosciences, Brunel UniversityUxbridge, UK; c Division of Metabolic and Vascular Health, Warwick Medical School, University of WarwickCoventry, UK

**Keywords:** G protein-coupled receptor, neurotensin receptor 1, apelin receptor, heterodimerization, resonance energy transfer, ERK _1/2_

## Abstract

Dimerization of G protein-coupled receptors (GPCRs) is crucial for receptor function including agonist affinity, efficacy, trafficking and specificity of signal transduction, including G protein coupling. Emerging data suggest that the cardiovascular system is the main target of apelin, which exerts an overall neuroprotective role, and is a positive regulator of angiotensin-converting enzyme 2 (ACE2) in heart failure. Moreover, ACE2 cleaves off C-terminal residues of vasoactive peptides including apelin-13, and neurotensin that activate the apelin receptor (APJ) and neurotensin receptor 1 (NTSR1) respectively, that belong to the A class of GPCRs. Therefore, based on the similar mode of modification by ACE2 at peptide level, the homology at amino acid level and the capability of forming dimers with other GPCRs, we have been suggested that APJ and NTSR1 can form a functional heterodimer. Using co-immunoprecipitation, BRET and FRET, we provided conclusive evidence of heterodimerization between APJ and NTSR1 in a constitutive and induced form. Upon agonist stimulation, hetrodimerization enhanced ERK_1/2_ activation and increased proliferation *via* activation of Gq α-subunits. These novel data provide evidence for a physiological role of APJ/NTSR1 heterodimers in terms of ERK_1/2_ activation and increased intracellular calcium and induced cell proliferation and provide potential new pharmaceutical targets for cardiovascular disease.

## Introduction

Apelin is an adipokine that exerts pleiotropic effects on the cardiovascular system. Emerging data have suggested that apelin is a positive regulator of angiotensin-converting enzyme 2 (ACE2) in heart failure *in vivo* 1. ACE2 is a negative regulator of the renin-angiotensin system (RAS), since it catalyses the conversion of angiotensin II (Ang II) to angiotensin 1-7 (Ang1-7) 2. Ang1-7 has been proposed to be one of key modulators of cardiovascular as well as renal function, thus counterbalancing the effects of Ang II and exerting a cardio-protective effect 3. Moreover, apelin is a substrate for catalytic ACE2 activity since it removes the C-terminal phenylalanine of the peptide *in vitro* 4.

Apelin exerts its affects by binding and activating the apelin receptor (APJ, gene symbol APLNR), a member of the GPCR super-family. GPCRs are central to many of the body's endocrine pathways and represent a major therapeutic target class 5. In terms of pharmacology, APJ is capable of binding a number of apelin isoforms, activate different classes of G protein α-subunits and subsequently induce several signalling pathways in cell-and tissue-specific manner 2,6. In accordance with other ‘promiscuous’ GPCRs, APJ is also capable of forming heterodimers with other GPCRs. For example, Chun *et al*., have shown using co-immunoprecipitation (Co-IP) and fluorescence resonance energy transfer (FRET) analysis that the Ang II receptors and APJ interacted physically 7. Similarly, Co-IP, co-localization and bioluminescence resonance energy transfer (BRET) assays confirmed the heterodimerization of APJ and kappa opioid receptor (KOR) 8. Interestingly, ACE2 also removed the C-terminal residue from the vasoactive peptide neurotensin *in vitro* 9. Neurotensin binds and activates the NTSR1 that is also a GPCR and shares 23% homology with APJ. Moreover, NTSR1 is capable of forming heterodimers with other GPCRs such as the dopamine D(2L) receptor 10 as well as homodimers 11. Given the similar mode of modification by ACE2 at peptide level, as well as the homology at amino acid level and the capability of forming dimers at receptor level, we have been suggested that APJ and NTSR1 can form a functional heterodimer.

In this study, we provide conclusive evidence for heterodimerization between NTSR1 and APJ using Co-IP, BRET and FRET *in vitro*. In addition, treatment of HEK293-APJ/NTSR1 cells with respective agonists significantly altered the phosphorylation status of ERK_1/2_ and increased intracellular calcium and promoted cell proliferation compared to cellular preparations where APJ or NTSR1 were expressed individually. Our findings provide a novel insight into a higher order of complexity on the signalling of apelin and neurotensin and provide a novel functional role for a new GPCR heterodimer.

## Materials and methods

### Plasmid constructs

pRluc-NTSR1: the full length human NTSR1 gene was amplified by PCR using the plasmid pcDNA3.1-NSTR1 (The Missouri S&T cDNA Resource Center, Rolla, MO, USA) as template with forward primer: 5′-ATAAGAATGCGGCCGCATGCGCCTCAACA GCTCCGCGCCG-3′ (Not I site), and reverse primer: 5′-GTTTAAACGGGCCCT CTAGACTAGTAC-3′ (Xba I site). The PCR product was inserted into the cloning sites (Not I/Xba I) of plasmid pRluc-N1 vector. The construction of pEGFP-APJ, pEYFP-APJ and pECFP-NTSR1 followed an identical methodology, with using the restriction enzyme sites EcoR I/BamH I for pEGFP-APJ or pEYFP-APJ and Hind III/Kpn I for pECFP-NTSR1. Restriction enzymes were purchased NEB (New England Biolabs, Hitchin, UK). All the constructs were verified by direct DNA sequencing. APJ shRNA plasmids (TRCN0000008097) and control shRNA plasmids (SHC002) were purchased from Sigma-Aldrich (St. Louis, MO, USA).

### Cell culture and transfection

Human umbilical vein endothelial cells (HUVEC; ATCC) were cultured in RPMI-1640 medium supplemented with 10% (v/v) foetal bovine serum (FBS; Life Technologies, Gaithersburg, MD, USA) at 37 with 5% CO_2_. Human Embryonic Kidney 293 (HEK293; ATCC) were cultured in MEM supplemented with 10% (v/v) FBS (Invitrogen Life Technologies). Transient transfection was performed with Lipofectamine 2000 (Life Technologies) according to the manufacturer's protocol. Following transfection, cells were maintained at 37°C with 5% CO_2_ for 6 hrs or overnight before replacing media for further 12–24 hrs before use.

### RNA extraction, cDNA synthesis and RT-PCR

Total RNA from HUVECs was extracted using the QIAGEN RNeasy kit (West Sussex, UK), DNase treated, and reverse transcribed into cDNA using a reverse transcriptase kit according to the manufacturer's instructions (Fermentas Life Sciences, York, UK). The sequences for the sense and antisense primers respectively were: NTSR1: 5′-CGCCAACAAGCTGACCGTCATG-3′ and 5′-GGCGATGACCA CTGCACGTAGG-3′ (157 bp); APJ: 5′-TCAGCAGCTACCTCATCTTC-3′ and 5′-AC TGCACCTTAGTGGTGTTC-3′ (231 bp). HEK293 cDNA was used as control for RT-PCR.

### Enzyme-linked immunosorbent assay (ELISA)

A day prior to transfection, HEK-293 cells were plated at a density of 2 × 10^5^ cells per well in 96-well plates. Transient transfection was performed with HA-APJ, Myc-NTSR1 and cotransfected HA-APJ and Myc-NTSR1 respectively, using Lipofectamine 2000 as previously described. After 24 hrs, cells were fixed using 4% paraformaldehyde in PBS (30 min. at 25°C). Fixed cells were washed three times with PBS, followed by incubation with blocking buffer (3% skimmed milk powder for 1 hr at 25°C). Cells were then incubated with the primary rabbit anti-HA antibody or anti-Myc antibody (1:500 in blocking buffer; Cell Signaling Technology, MA, USA) overnight at 4°C, washed three times with PBS and incubated with a peroxidase conjugated goat/rabbit secondary antibody (1:2000) in DMEM for 1 hr at 37°C. The final substrate (200 μl) 3,3′,5,5′-tetramethylbenzidine (Sigma-Aldrich, St. Louis, MO, USA) was added and incubated for 30 min. at 37°C and the enzymatic reaction stopped with the addition of 50 μl of 2 N H_2_SO_4_. The colorimetric reaction was measured using an iMark™ Microplate reader (Bio-Rad, Hercules, CA, USA) at 450 nm wavelength.

### Confocal laser scanning microscopy

HEK293 cells grown on coverslips were transiently transfection with pEGFP-APJ and pcDNA3.1-NTSR1 and fixed after 24 hrs as previously described. Non-specific binding was blocked by incubation with 3% bovine serum albumin (BSA) in PBS-Triton X-100 (0.01%) for 1 hr at room temperature. Cells were washed three times with PBS then incubated overnight with anti-NTSR1 monoclonal antibody (1:200) in PBS at 4°C. Cells were washed three times with PBS followed by incubation for 1 hr at room temperature with Rhodamine-conjugated AffiniPure goat anti-rabbit IgG (1:400). After final washes with PBS, cells were observed using a laser scanning confocal microscope (oil-immersion x40 objective; TSC SP5, Leica, Germany).

### Western blotting

Cells were washed with cold PBS and lysed in lysis buffer (50 mM Tris–HCl, pH 7.4, 150 mM NaCl, 1 mM EDTA, 10 mM MgCl_2_, 1 mM DTT, 1 mM PMSF, 1 μg/ml leupeptin, 2 μg/ml aprotinin, 1.4 μg/ml pepstatin A and 0.5% (v/v) NP-40). 10 μg of cell lysates were separated by 10% SDS-PAGE followed by transfer to PVDF membrane. Samples were visualized as previously described 12. The proteins of interest were probed with primary antibodies described above.

### Co-immunoprecipitation and western blotting

Co-immunoprecipitation was performed as previously described 7. HEK293 cells were cotransfected with HA-APJ and Myc-NTSR1 or control vector. After 48 hrs, the cells were lysed in 800 μl lysis buffer (120 mM NaCl, 0.5% NP-40, 100 mM NaF, 50 mM Tris–Cl, pH 8.0). Following centrifugation at 4°C for 15 min. at 16,000 g, 500 μl of whole cell lysates were incubated with 20 μl HA-conjugated Sepharose beads (Cell Signaling Technology) for 4 hrs with gentle rotation at 4°C. The beads were washed four times with cell lysis buffer. Protein complexes were eluted with 2 × SDS-PAGE sample buffer and anti-Myc and anti-HA immunoreactivity was detected by western blotting. To monitor the protein expression level, 10% of total cell lysates of each sample were used for western blots.

### BRET measurements

To monitor the constitutive interactions between NTSR1 and APJ, saturation assays were performed: HEK293 cells were transfected with pRluc-NTSR1 and pEGFP-APJ plasmids at a ratio of 1:0.5, 1:1, 1:1.5, 1:2, 1:2.5, 1:3 to express a constant amount of donor-labelled protein with increasing amounts of acceptor-labelled protein to be assayed 13. About 24 hrs after transfection, cells were trypsinised and plated on a 96-well microplate for 24 hrs in HEPES-buffered phenol red-free medium (Invitrogen, Life Technologies). Cells were then washed and re-suspended in 50 μl D-PBS. 5 μM of Coelenterazine h (Promega, Southampton, UK) was added for BRET measurements using the Tristar LB941 plate reader (Berthold Technologies, Bad Wildbad, Germany) with two filter settings (Rluc filter, 400–475 nm; and EGFP filter, 500–550 nm). At the same time, pRluc and pEGFP-APJ, pRluc-NTSR1 and pEGFP were detected to eliminate the interference. BRET signal was detected and demonstrated by mBRET (mBRET = BRET signal × 1000).

To monitor the induced interaction between NTSR1 and APJ, BRET signal was measured as described above with slight modification: briefly, cells were transiently transfected with pRluc-NTSR1 and/or pEGFP-APJ and stimulated with Neurotensin (NT) (10^−7^ M) or apelin-13 (10^−7^ M) (Phoenix Pharmaceuticals, Belmont, USA) for 15 min. Following washes, cells were re-suspended in D-PBS and 5 μM Coelenterazine h was added for BRET measurements.

### FRET imaging

FRET was also used to explore further the heterodimerization between NTSR1 and APJ in living cells. The fluorescence of FRET channel (raw FRET) comprises of the actual FRET and also the bleed-through from the fluorescent proteins, namely, raw FRET = FRET+(A*receptor)+(B*donor), so this FRET signal was corrected using Coefficient A and Coefficient B, which describe bleed through correcting the raw FRET image using the sensitized emission algorithm 14. To obtain these coefficients, cells were transfected with pEYFP and pECFP individually. After obtaining these coefficients, FRET was performed. Donor plasmid pCFP-NTSR1 and receptor plasmid pEYFP-APJ were cotransfected into HEK293 cells, in addition, pECFP-NTSR1 and pEYFP, pECFP and pEYFP-APJ were also cotransfected into HEK293 cells as a negative control. After 12–24 hrs, FRET signal was detected.

### Knockdown of APJ in HUVEC

HUVECs were plated at 70–80% confluency before transfection. Cells were transfected with APJ shRNA and control shRNA plasmids using Lipofectamine 2000 according to the manufacturer's instructions. About 12 hrs after transfection, the cells were screened by puromycin and after 24 hrs, cells were washed twice with PBS, detached with trypsin containing 0.25% EDTA and then distributed in a 6-well plate for 24 hrs. Then the cells were stimulated with NT or apelin-13 at 10^−7^ M final concentration in serum free medium. Protein expression of interest was assessed by Western blot analysis.

### Calcium fluorescence measurement

Calcium signals were detected by using Fluo-4NM Calcium Assay Kits (Invitrogen, Carlsbad, California, USA) following manufacturer's instructions. HEK293-APJ, HEK293-NTSR1 and HEK293-APJ/NTSR1 were plated at 5 × 10^4^ cells per well in a poly-d-lysine-coated black 96-well plate and grown overnight. Fluo-4NW assay kit (Invitrogen) was then used. Briefly, 100 μl Fluo-4NW working solution was added to each well. Cells were incubated at 37°C in 5% CO_2_ humidified chamber for 30 min., then at room temperature for an additional 30 min. The plates were then washed three times with an assay buffer to remove excess dye. Subsequently, all cells were stimulated with apelin-13 or neurotensin and fluorescence were immediately measured with the TriStar LB 941 Multimode Microplate Reader (Berthold Technologies); DLR ready and appropriate for excitation at 485 nm and emission at 525 nm. The calcium ratio was measured by using the fluorescence value after adding the agonist taking away fluorescence value before adding agonist divided by the fluorescence value before adding the agonist. The peak increase in fluorescence counts was recorded for each well. All the experiments were repeated in triplicates.

### NFAT luciferase reporter assays

For the nuclear factor of activated T-cells (NFAT) luciferase reporter assay, cells stably expressing APJ, NTSR1 or APJ/NTSR1 were cotransfected with the DNA of pNFAT-Luc (nuclear factor of activated T cells tagged with luciferase) (PathDetect, Stratagene) which contain firefly luciferase and pRL-Tk (Promega). About 24 hrs after transfection, the cells were serum starved and stimulated with NT or apelin-13 at 10^−7^ M final concentration for 6 hrs prior to harvest. Relative luminescence units (RLU) for firefly and renilla luciferase were determined using Dual-Glo luciferase assay kits (Promega) according to the manufacturer's instructions.

### Cell proliferation

HEK293 cells were transiently transfected with pcDNA3.1-NTSR1 and/or pcDNA3.1-APJ and cultured in 96-well plates (1 × 10^4^ cells per well) in complete media treated with NT or apelin-13 at 10^−7^ M final concentration for 36 hrs. Cell proliferation was measured according to EnoGene Cell™ Counting Kit-8 (Promega) with iMark™ Microplate reader (Bio-Rad).

### Statistical analysis

Data are expressed as mean ± SEM. Statistical comparisons were performed with the Student's *t*-test. In the case of multiple group comparisons, anova was adopted. Differences among means were considered significant when *P* < 0.05.

## Results

### The endogenous expression of APJ and NTSR1 in HUVECs

**Figure 1:**
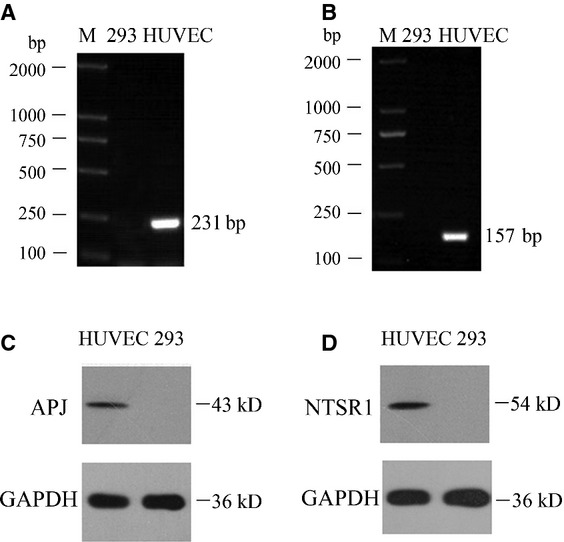
APJ and NTSR1 expression in HUVEC and HEK293 cells. RT-PCR revealed that HUVECs but not HEK293 cells express APJ and NTSR1 (**A, B**). Similar expression was noted for both receptors at protein level as they were detected by western blot (**C, D**). Lane M: DNA Ladder, Lane 293: Untransfected HEK293 cells.

RT-PCR confirmed the expression of APJ and NTSR1 as 231 and 157 bp products respectively in HUVECs but not in HEK293 cells (Fig.1A and B). Both receptors are also expressed as proteins at 54 kD for APJ and a 43 kD for NTSR1 (Fig.1C and D) in HUVECs. Similarly, no apparent detection of either of the receptors was evident in HEK293 cells.

### NTSR1 and APJ are capable of co-expression and co-localization

**Figure 2:**
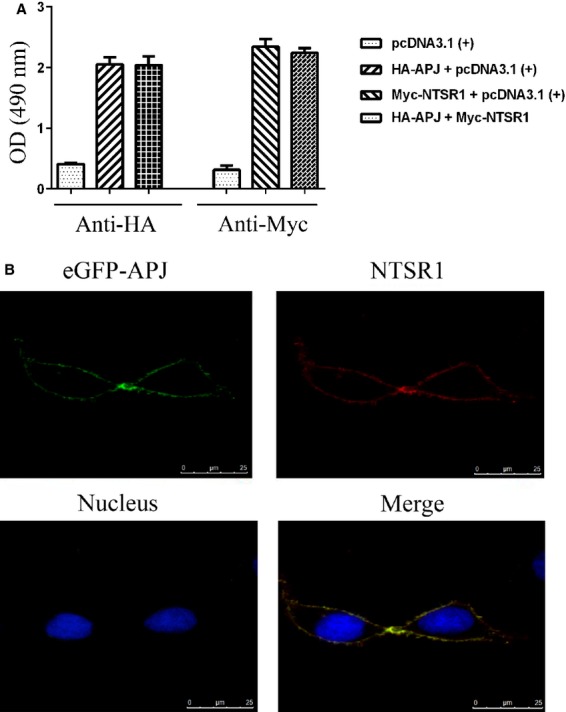
Co-expression and co-localization of APJ and NTSR1. Determination of cell surface expression of HA-APJ and Myc-NTSR1 by ELISA on intact cells using anti-HA and anti-Myc antibody. Plasmids expressing HA-tagged APJ or Myc-tagged NTSR1 were transfected alone or in combination, as illustrated. Values were presented as mean ± SEM of triplicates (**A**). pEGFP-APJ (green) and pcDNA3.1-NTSR1 were transfected into HEK293 for 24 hrs, pcDNA3.1-NTSR1 was immunostained with Rhodamine (red) and the cell nuclei were stained with dye-DAPI (blue). Immunofluorescence was measured with a Leica fluorescent microscope (**B**).

We examined whether of NTSR1 and APJ can be co-expressed. Cell surface expression of HA-APJ and Myc-NTSR1 were measured by ELISA using anti-HA and anti-Myc antibody in HEK293 cells expressing HA tagged APJ alone, Myc tagged NTSR1 alone or co-expressing HA-APJ and Myc-NTSR1. Based on the absorbance readings co-expression of NTSR1 and APJ does not alter their expression levels (Fig.2A). To study the co-localization of NTSR1 and APJ, immunofluorescence analysis was carried out in HEK293 cells cotransfected with plasmids expressing NTSR1 and APJ. Confocal laser scanning microscopy revealed a high degree co-localization of NTSR1 and APJ, predominantly around the plasma membrane, consistent with their function as GPCRs as well as their potential capacity to form heterodimers (Fig.2B).

### Assessment of NTSR1 and APJ heterodimerization by co-immunoprecipitation, BRET and FRET

**Figure 3:**
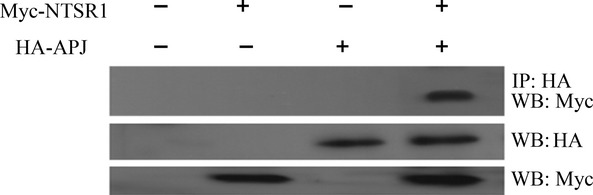
Assessment of dimerization between APJ and NTSR1 using co-IP. HEK293 cells were mock transfected or transfected with expression vectors of both HA-APJ and Myc-NTSR1 fusion proteins (cotransfected) or with either vector alone. Confirmation of the expression of appropriate constructs was obtained by immunoblotting cell lysates with either anti-Myc or anti-HA antibodies (lower panel). The cell lysates were subsequently immunoprecipitated (IP) with anti-HA antibody and immunoblotted with anti-Myc antibodies (upper panel) to demonstrate the dimerization between APJ and NTSR1.

**Figure 4:**
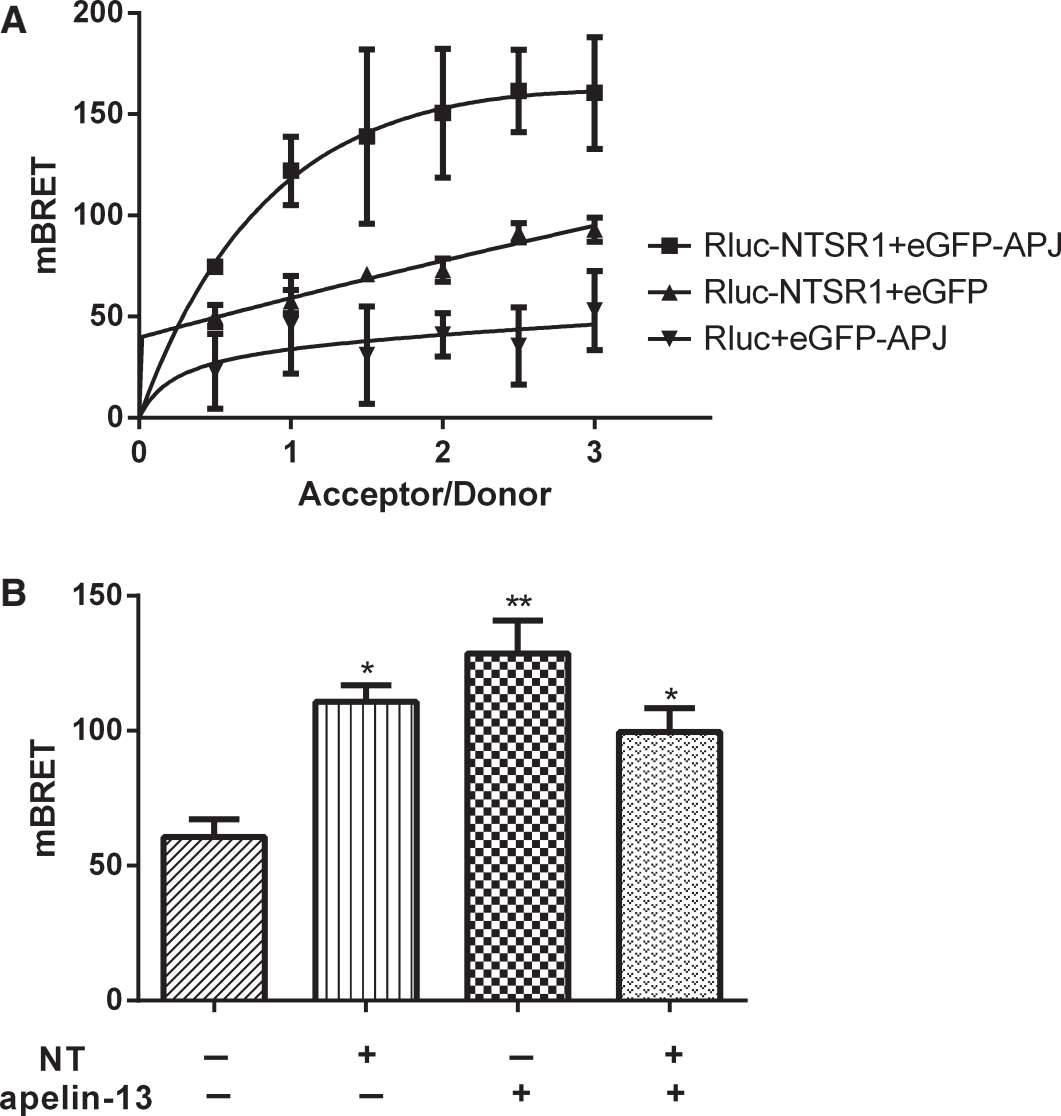
HEK293 cells were transfected with a constant amount of pRluc-NTSR1 with increasing amounts of pEGFP-APJ by 1:0.5, 1:1, 1:1.5, 1:2, 1:2.5, 1:3. BRET ratio demonstrated as mBRET (Mbret = BRET × 1000). All the results were expressed as mean ± SEM of at least six independent assays (**A**). HEK293 cells were transiently cotransfected with pRluc-NTSR1 and pEGFP-APJ (1:1.5), and after 24 hrs upon addition of substrate Coelenterazine-h, cells were treated with or without NT (10^−7^ M) and/or apelin-13 (10^−7^ M) for 15 min. and BRET signals were analysed (**B**). All the results were expressed as mean ± SEM of at least six independent assays. *P < 0.05 *versus* control; **P < 0.01 *versus* control.

Co-IP analysis of HA-APJ/Myc-NTSR1 in cotransfected HEK293, revealed that only when both receptors were co-expressed, immunoreactive bands against the anti-HA and anti-Myc antibodies were detected; indicative of a complex formation (Fig.3). BRET studies were carried out to study further the heterodimerization between NTSR1 and APJ. In saturation assays, HEK293 cells cotransfected with Rluc-NTSR1 and EGFP-APJ result in similar BRET signals to saturation curves of positive control. However, the negative controls result in BRET signals that increase with increasing concentrations of acceptor-labelled protein in a quasi-linear manner (Fig.4A), indicating a constitutive interaction between NTSR1 and APJ.

Furthermore, treating co-expressed receptors with NT (10^−7^ M) and/or apelin-13 (10^−7^ M), the BRET signals were significantly up-regulated when compared to control unstimulated cells (Fig.4B). These data demonstrate an induced interaction between NTSR1 and APJ upon agonist stimulation. When both agonists were used for treatment, there was no additive effect.

**Figure 5:**
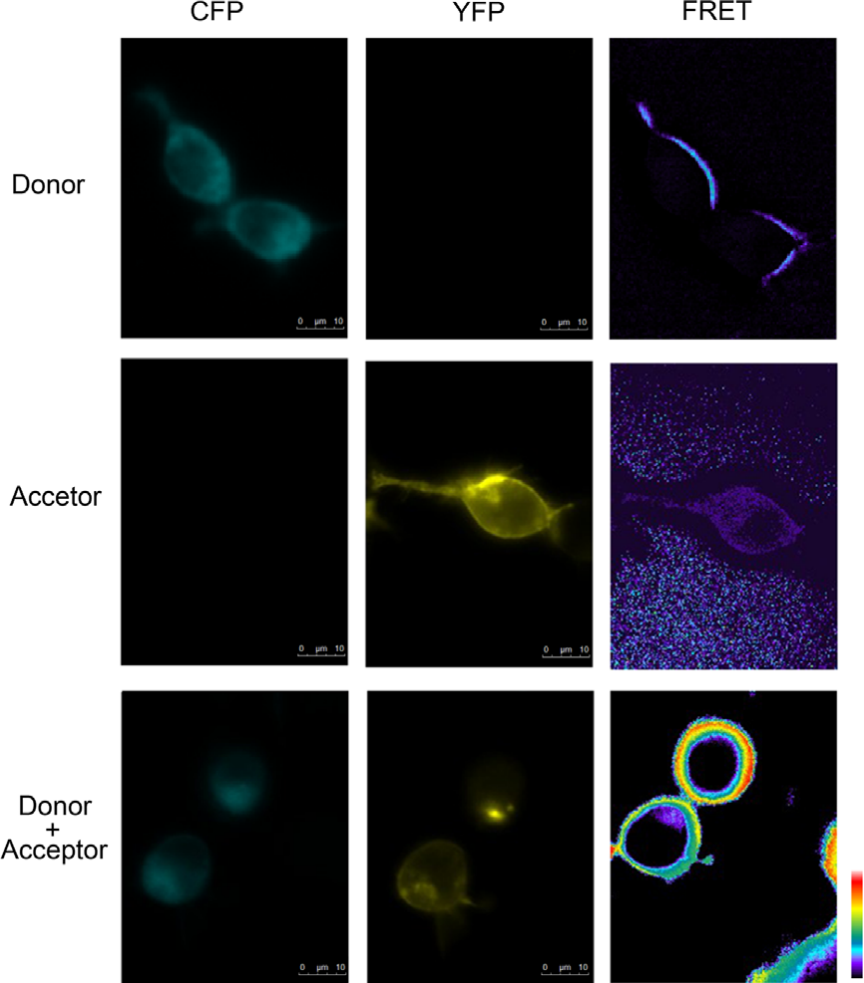
Assessment of dimerization between APJ and NTSR1 using FRET assay. HEK293 cells were cotransfected with pCFP-NTSR1 and/or pYFP-APJ. Fluorescent images acquired using the indicated filters 24 hrs after transfection. Pseudocolor FRET images were generated by MetaFluor 7.0 Software, with the lowest FRET intensity in black and the highest FRET intensity in red.

FRET was carried out to provide further evidence for the heterodimerization between NTSR1 and APJ in living cells. The fluorescence measurements resulted in the following parameters: Coefficient A and Coefficient B is 0.39 and 0.55, respectively. No FRET signal was detectable in pECFP-NTSR1 and pEYFP-APJ transfected alone. However, a notable FRET signal detected only in pECFP-NTSR1 and pEYFP-APJ cotransfected in HEK293 cells (Fig.5), corroborating the Co-IP and BRET findings.

### NT and Apelin-13 induced ERK_1/2_ activation in the HEK293-APJ/NTSR1 cells

**Figure 6:**
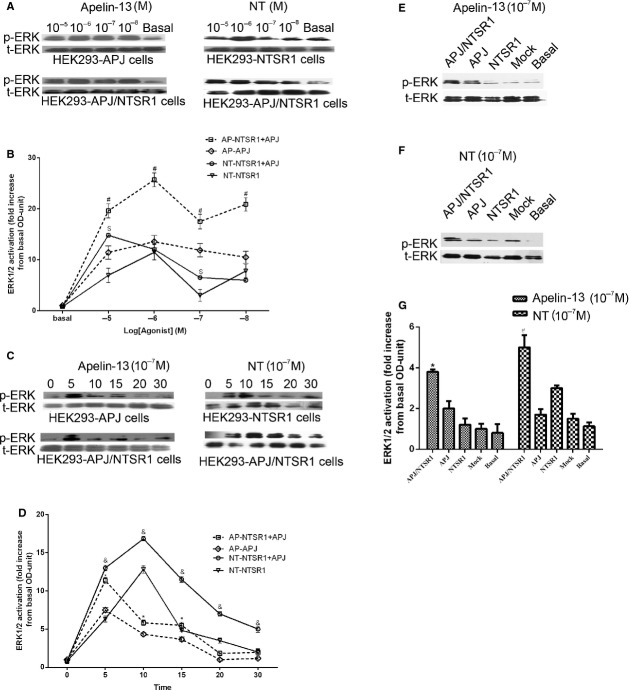
The effect of APJ and NTSR1 heterodimerization on ERK_1/2_ phosphorylation. Dose-dependent ERK_1/2_ phosphorylation HEK293-APJ or HEK293-NTSR1 cells (upper panels) and HEK293-APJ/NTSR1 cells (lower panels) treated with Apelin-13 or NT (**A**). Summary of the dose–responses of apelin-13 and NT on ERK_1/2_ activation (**B**). HEK293-APJ or HEK293-NTSR1 cells (Upper panels) and HEK293-APJ/NTSR1 cells (Lower panels) were treated with apelin-13 or NT (10^−7^ M) from 5 to 30 min. (**C**). Quantification of all time-dependent treatments in HEK-293 cells expressing APJ, NTSR1 or a combination of both (**D**). Effect of apelin-13 or NT (10^−7^ M) stimulation on ERK_1/2_ phosphorylation in HEK293, HEK293-APJ, HEK293-NTSR1 or HEK293-APJ/NTSR1 cells (**E, F**). Quantification ERK_1/2_ phosphorylation corrected over total ERK of apelin-13 or NT treatments in HEK-293 cells expressing APJ, NTSR1 or a combination of both (**F**). All the results were expressed as mean ± SEM. *P < 0.05 *versus* Apelin-13 stimulated HEK293-APJ groups; ^&^*P* < 0.05 *versus*NT stimulated HEK293-NTSR1 group.

Dose-and time-dependent effects of NT and Apelin-13 on ERK_1/2_ activation were then determined. Both ligands were able to induce phosphorylation of ERK_1/2_ upon treatment of HEK293 cells transfected with their concomitant receptors or when the receptors were co-expressed (Fig.6A). Interestingly, ERK_1/2_ activation was significantly higher in HEK293-APJ/NTSR1 group than that in HEK293-APJ group treated with apelin-13 (10^−8^–10^−5^ M) or HEK293-NT group treated with NT (10^−8^–10^−5^ M). ERK_1/2_ reached maximal phosphorylation upon treatment with apelin-13 at 10^−6^ M or NT at 10^−5^ M (Fig.6B). With regard to time-dependent ERK_1/2_ activation, HEK293-AJP, HEK293-NTSR1 and HEK293-APJ/NTSR1 cells were treated with 10^−7^ M NT or Apelin-13 for 0, 5, 15, 20 and 30 min. There was a significant activation of ERK_1/2_ for apelin-13 treated HEK293-AJP and HEK293-APJ/NTSR1 cells peaking at 5 min. after stimulation (Fig.6C and D). HEK293-NTSR1 and HEK293-APJ/NTSR1 cells treated with NT demonstrated maximum phosphorylation of ERK_1/2_ at 10 min. after treatment (Fig.6C and D). In both cases, the degree of phosphorylation was greater when NTSR1 and APJ were co-expressed.

To eliminate the possibility that enhanced ERK activation was caused by cross-agonism or other signalling effects, we tested the effects of apelin-13 (10^−7^ M) on HEK293 cells expressing NTSR1 and the effects of NT (10^−7^ M) treatment on HEK293 cells expressing APJ. The results show that the increased ERK_1/2_ phosphorylation was only observed in the cells expressing both receptors (Fig.6E–G), and there is no apparent interaction between apelin-13 with NTSR1, and NT with APJ. Collectively, these indicate that there is no cross-agonism between the two GPCRs and their cognate ligands.

### RNAi to APJ compromised ERK_1/2_ activation in HUVECs

**Figure 7:**
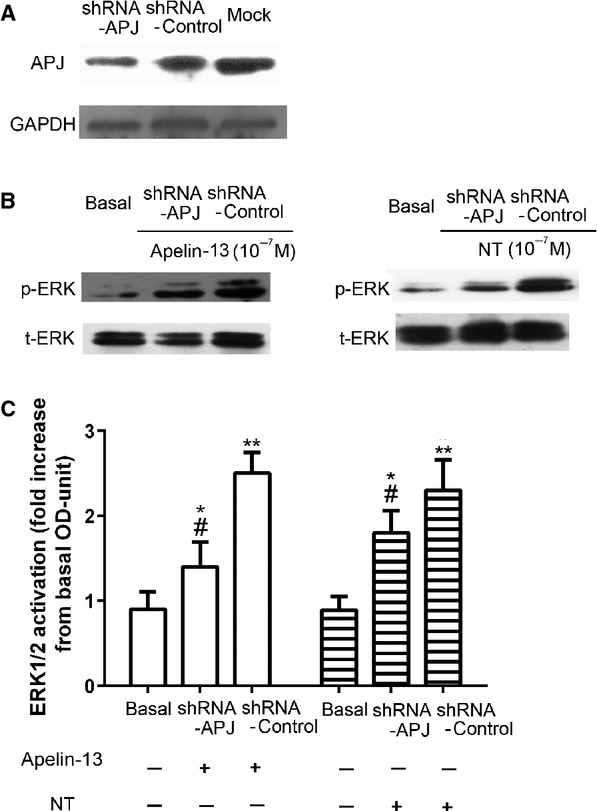
Effects of Knockdown APJ on ERK_1/2_ phosphorylation in HUVECs APJ. shRNA efficiently knocks down APJ expression in HUVEC cells. HUVECs were transfected with APJ shRNA or control shRNA. 48 hrs after transfection, cell lysates were subjected to western blot and incubated with antibodies against APJ. GAPDH was used as the loading control (**A**). HUVECs were transfected as previous described and treated with apelin-13 or NT at 10^−7^ M for 15 min. respectively. Cell lysates were subjected to western blot and incubated with antibodies against phosphor-ERK and total ERK. HUVECs without shRNA transfection and agonist stimulation were used as basal control. Quantification of the effects of APJ knockdown on ERK_1/2_ phosphorylation corrected over total-ERK (**C**). All the results were expressed as mean ± SEM. *P < 0.05 *versus* basal group; **P < 0.01 *versus* basal group; ^#^*P* < 0.05 *versus* Control shRNA group.

HUVECs were transiently transfected with shRNA APJ and shRNA Control as experimental group and control group respectively. Western blot demonstrated that the protein expression in shRNA APJ group was lower than that in shRNA group and basal group, while there were no significant differences between shRNA Control group and basal group (Fig.7A), demonstrating that shRNA APJ can effectively decrease the APJ expression at protein level. Then, shRNA APJ group and shRNA group were stimulated with apelin-13 or NT, while the basal group was constituted of unstimulated cells. When compared to shRNA Control group, ERK_1/2_ activation in shRNA APJ group was significantly decreased by apelin-13 stimulation. Interestingly, decrease in APJ expression also resulted to a weaker ERK_1/2_ activation after NT stimulation (Fig.7B and C). This effect could be because of the heterodimerization of NTSR1 and APJ, involved in this particular signalling cascade.

### Heterodimerization of APJ and NTSR1 enhances NT or apelin-13 induced cell proliferation

**Figure 8:**
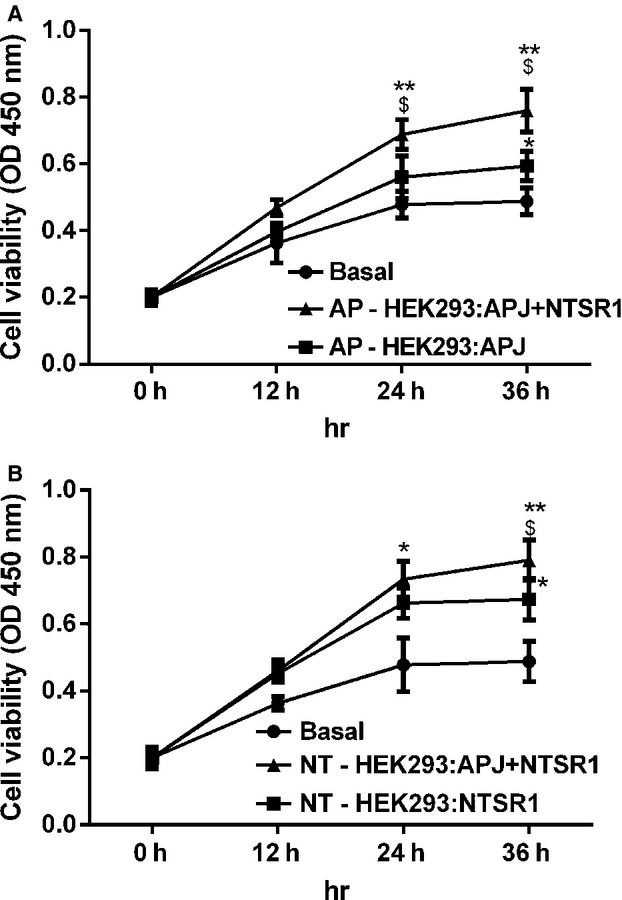
Effects of heterodimerization of APJ and NTSR1 on HEK293 cell proliferation. HEK293-APJ cells and HEK293-APJ/NTSR1 cells were treated with apelin-13 (10^−7^ M) (**A**). HEK293-NTSR1 cells and HEK293-APJ/NTSR1 cells were treated with NT (10^−7^ M) for 36 hrs (**B**). The cell proliferation was measured by CCK assays. All the results were expressed as mean ± SEM.*P < 0.05 *versus* basal group, **P < 0.01 *versus* basal group, ^$^*P* < 0.05 *versus* Single (apelin-13 or NT) stimulated groups.

Cell viability was measured from 0 to 36 hrs in treated cells with NT or apelin-13 (10^−7^ M). Both NTSR1 and APJ stimulation significantly induced cell proliferation (Fig.8). Interestingly, the cell viability of HEK293-APJ/NTSR1 group was also higher when compared to HEK293-APJ or HEK293-NTSR1 group alone treated with apelin-13 or NT respectively (Fig.8A and B). These data demonstrate that activation of APJ/NTSR1 heterodimers could increase HEK293 cell proliferation.

### NTSR1 and APJ heterodimerization enhances the Gq α-subunits activation

**Figure 9:**
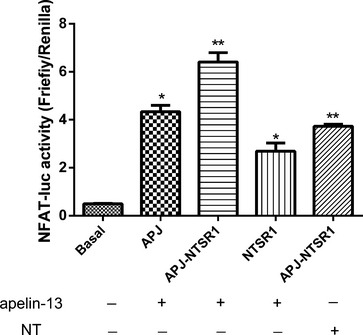
The effect of dimerization between APJ and NTSR1 on Gαq activation. NFAT activities of HEK293-APJ, HEK293-NTSR1 and HEK293-APJ/NTSR1 cells stimulated with apelin-13 (10^−7^ M) or NT (10^−7^ M). All the results were expressed as mean ± SEM. *P < 0.05 *versus* basal group; **P < 0.01 *versus* basal group.

We dissected further the effects of dimerization on downstream signal transcription by measuring nuclear factor of activated T-cells response element (NFAT) using a double luciferase reporter assay. Compared with control cells (basal), HEK293-AJP and HEK293-NTSR1 cells, the activity of NFAT-luc was significantly increased in HEK293-APJ/NTSR1 cells. HEK293 cells transfected only with AJP or NTSR1, also demonstrated a moderate but significant induction of NFAT luciferase activity compared to basal unstimulated levels (Fig.9). These results imply that heterodimerization of NTSR1 and APJ significantly up-regulates NFAT activity, which involves the Gαq pathway.

### Heterodimerization of NTSR1 and APJ increase intracellular Ca^2+^ upon agonist stimulation

**Figure 10:**
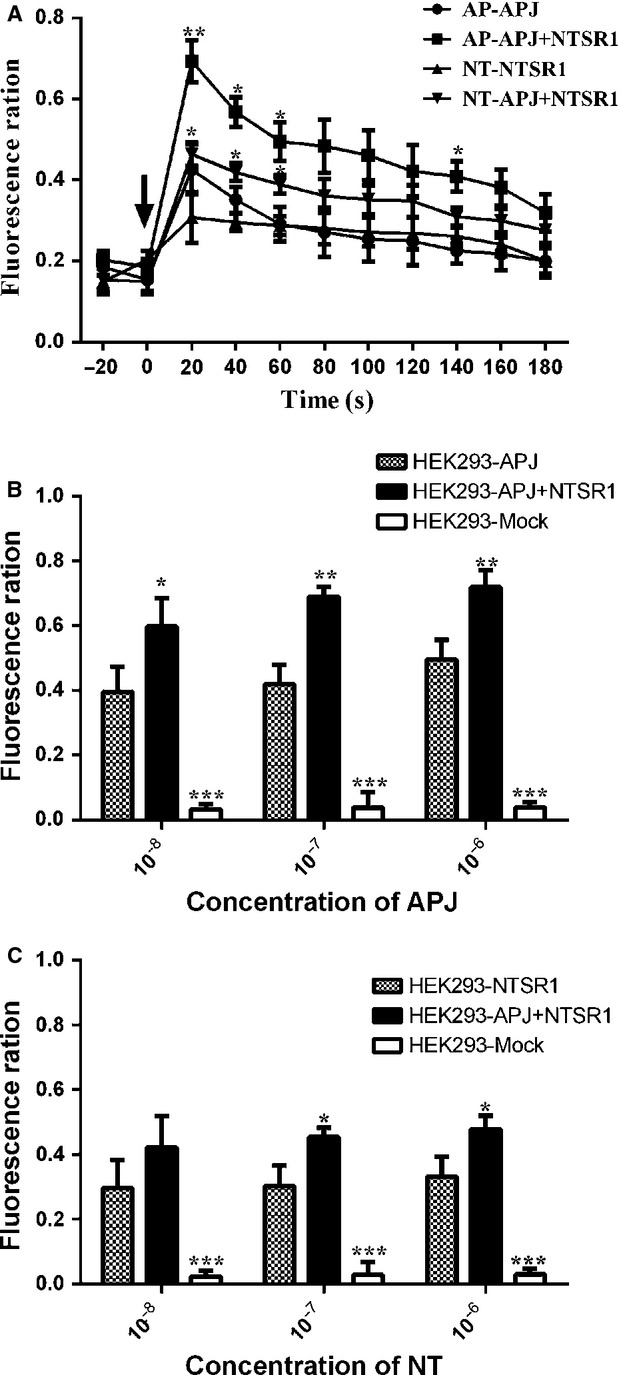
The effects of co-expression of APJ and NTSR1 on intracellular Ca^2^ ^+^ . HEK293 cells expressing APJ, NTSR1 or APJ with NTSR1 cells were plated in a poly-d-lysine coated 96-well plate, incubated overnight, and assayed for a calcium response to apelin-13 or NT using the Fluo-4 Direct™ assay. Calcium fluorescence responses kinetics for apelin-13 or NT stimulation was expressed in terms of fluorescent change over background (**A**). Arrows mark the time of compound addition. (**A**) Each fluorescent trace represents the calcium response resulting from a dose of a single concentration (10^−7^ M) of apelin-13 or NT added to a single well. The maxima of time periods (0–180 sec.) of each trace were used dose–response curve construction for apelin-13 (**B**) or NT (**C**) responses, respectively. Data represented as mean ± SEM of three measurements from three independent experiments. *P < 0.05, ***P < 0.01, ***P < 0.001 compared to single transfected groups.

As indicated, NTSR1 and APJ heterodimers can induce NFAT activity *via* a Gαq pathway. To investigate this further, we evaluated changes of calcium ions in cells after stimulation by apelin-13 and NT at 10^−7^ M. Treatment of HEK293-APJ/NTSR1 cells with apelin-13 resulted in significant up-regulation of fluorescence signal compared with that of in HEK293-APJ cells. A similar effect was also noted between HEK293-APJ/NTSR1 cells and HEK293-NTSR1 cells treated with NT (Fig.10A). This effect was observed on a wide range of apelin-13 or NT concentrations: from 10^−8^ to 10^−6^ M (Fig.10B and C).

These results provide conclusive evidence that heterodimerization of NTSR1 and APJ could increase the level of calcium upon both agonists stimulation. Since the Gαq activation leads to the increase in intracellular calcium concentration, these results further strengthen the hypothesis that the induction of NFAT activity relies on the activation of this G protein α-subunit.

## Discussion

A plethora of studies have provided evidence for the interaction and cross-talk of GPCRs, namely, the existence of homodimeric or heterodimeric or even higher structure oligomers that are dependent, or independent of agonist stimulation, which form new receptor complexes and give receptors unique characteristics that exhibit functional properties distinct from monomeric receptors involving agonist recognition 15, G-protein preferences 16 and signalling. In particular, the apelin/APJ system plays a fundamental role in the occurrence and development of cardiovascular diseases 2. In this study, we confirmed a high degree of co-localization of APJ with NTSR1, predominantly on the plasma membrane of HEK293 cells. We have also conclusively demonstrated that APJ and NTSR1 can exist as heterodimers in constitutive and induced form by Co-IP, BRET and FRET. Upon agonist stimulation, heterodimerization increased ERK_1/2_ activation, intracellular calcium and NFAT activity and induced cell proliferation.

In our *in vitro* system, ERK_1/2_ activation was significantly enhanced in HEK293-APJ/NTSR1 cells compared to that in HEK293-APJ or HEK293-NTSR1 cells after treatment with agonists. This effect was also mirrored when cell proliferation and intracellular calcium was measured. Recent studies have shown that apelin-13 increased vascular smooth muscle cell proliferation by up-regulating the expression of cyclin D1, which is involved in an ERK-dependent activation of Jagged-1/Notch3 signalling 2,17. In the same study, apelin-13 was able to induce phosphorylation of ERK_1/2_ in a dose-and time-dependent manner, thus corroborating our findings. Moreover, the proliferative effect of apelin has been well documented in other cells such as retinal endothelial cells 18, human osteoblasts and gastric cells 19. Interestingly, down-regulation of APJ expression dampens not only apelin-13 but also NT induced ERK_1/2_ activation. This finding was consistent with the increase in ERK_1/2_ phosphorylation in HEK293 cells co-expressing APJ and NTSR1. There was no evidence of cross-agonism between the APJ and NTSR1 when activated by NT and apelin-13 respectively.

Apelin/APJ and NT/NTSR1 system are co-expressed in a wide range of tissues, playing a role in neuroprotection and involved in neuropsychiatric and cardiovascular disease 20,21. APJ and NTSR1 could exist as heterodimers in HUVECs therefore activation of the dimeric GPCR complex might also mediate endothelial cell proliferation and vascular functions such as angiogenesis. For example, using HUVECs as a cellular model, Herr *et al*. have demonstrated the potential capacity of angiotensin II in influencing angiogenesis by the regulation of angiogenesis-associated genes *via* AGTR1^22^. However, the proliferative effects of this heterodimer have a scope beyond vasculature, since injection of apelin into the ischaemic myocardium facilitates neovascularization in the peri-infarct area 23.

We propose that activation of ERK_1/2_, followed by increased cell proliferation in the heterodimeric state is primarily regulated by activation of the Gq α-subunit and involves increase in intracellular calcium. The receptors investigated in this study belong to GPCRs A group and share very similar G-protein coupling preferences (*i.e*. Gαi/o and Gαq) to activate ERK_1/2_ 24,25. In a monomeric state, NTSR1 can activate multiple G-proteins Gαi, Gαs and Gαq, but mainly Gαq, whereas APJ can activate mainly Gαi and partially Gαq. Upon heterodimer formation between APJ and NTSR1, Gαq is preferentially activated. This is in agreement with a previous study of the cannabinoid (CB1) receptor and the dopamine D2 receptor (D2) heterodimer. Co-expression of the D2 receptor was sufficient to switch CB1 from Gαi to Gαs coupling, even in the absence of a D2 agonist 26.

In an elegant study by White *et al*., it was shown that when NTSR1 exist as a monomer, it can activate readily Gαq, whereas upon receptor homodimerization the catalysis of nucleotide exchange occurs less efficiently 16. The authors concluded that NTSR1 homodimerization is not required for G protein activation. In our study, there is an augmentation of signalling responses upon hetero-but not homodimerization. It is possible that the APJ/NTSR1 heterodimer does not really inhibit any of the G protein α-subunits binding sites. Future studies using photoaffinity labelling, or co-IP can provide a further insight into the binding capacity of the heterodimer with various G proteins.

Collectively our data provide evidence for a novel signalling pathway involving the formation of a new GPCR heterodimer. The implications of this finding can be of increasing significance for the regulation of apelin/APJ system at cardiac level and beyond. For example, apelin-deficient hearts in mice express significantly less ACE2 and in wild-type mice activation of apelin-13/APJ system induced ACE2 expression in cardiomyocytes 1. In another study, APJ^−/−^ embryos surviving to later stages demonstrated incomplete vascular maturation because of a deficiency of vascular smooth muscle cells and impairment of the ventricular wall development 27. Recent studies demonstrated that GPCR heterodimers have tremendous physiological relevance, making them attractive drug targets in heath and disease 28. For instance, μ-opioid receptor and α 2A-adrenergic receptor can interact and affect the physiological processes such as neurogenesis 29. In addition, angiotensin II type 1 receptor and bradykinin B2 receptor dimers play a critical role in preeclampsia because of increase in angiotensin activity induced by the dimers 30. Based on our findings we believe APJ-NTSR1 dimers may be involved in RAS, regulating cardiovascular physiology. For example, in cases of a deficiency of apelin-13/APJ, heterodimerization with NTSR1, or treatment with NT might exert a compensatory response and become an attractive therapeutic target. Future studies should concentrate on elucidating the heterodimerization of AJP/NTSR1 in cardiac tissues and endothelial cells, and use them as experimental models to delineate their precise role in cardiac physiology.
